# Characterization of Large Extracellular Vesicles Released by Apoptotic and Pyroptotic Cells

**DOI:** 10.3390/ijms27020976

**Published:** 2026-01-19

**Authors:** Delaram Khamari, Nora Fekete, Ririka Tamura, Raeeka Khamari, Agnes Kittel, Bence Nagy, Luigi Menna, Zsuzsanna Darula, Alicia Galinsoga, Eva Hunyadi-Gulyas, Maximilien Bencze, Edit I. Buzas

**Affiliations:** 1 Institute of Genetics, Cell- and Immunobiology, Semmelweis University, 1085 Budapest, Hungary; frescodela@gmail.com (D.K.);; 2 HUN-REN-SU Translational Extracellular Vesicle Research Group, 1085 Budapest, Hungary; 3 Henri Warembourg Faculty of Medicine, Lille University Hospital (CHU), 59037 Lille, France; 4 HUN-REN (Hungarian Research Network), Institute of Experimental Medicine, 1083 Budapest, Hungary; 5 Single Cell Omics Advanced Core Facility, Hungarian Centre of Excellence for Molecular Medicine, 6728 Szeged, Hungary; 6 Proteomics Research Group, Core Facility, HUN-REN Biological Research Centre, 1083 Szeged, Hungary; 7 U955-IMRB, Inserm, UPEC, ENVA, EFS, Team Relaix, Biology of the Neuromuscular System, 94000 Créteil, France

**Keywords:** extracellular vesicles, apoptosis, pyroptosis, cell death

## Abstract

Extracellular vesicles (EVs) are emerging as key factors in maintaining cellular homeostasis, critical mediators of intercellular communication, potential biomarkers, and therapeutic tools. While small EVs have been extensively characterized, the molecular signatures of large EVs (including those generated during regulated cell death pathways) remain poorly defined. Here, we investigated the characteristics of large EVs released during apoptosis and pyroptosis by human monocytic cell lines (THP-1 and U937). Apoptosis was induced by staurosporine and blocked using the pan-caspase inhibitor Q-VD-OPh, whereas pyroptosis was triggered by LPS/nigericin and inhibited with a selective NLRP3 inhibitor. We found that both forms of regulated cell death markedly enhanced the release of large EVs. Both apoptotic and pyroptotic large EVs showed increased Annexin V binding and decreased CD9 expression compared with those released by healthy cells. Large EVs derived from apoptotic and pyroptotic cells exhibited distinct proteomic profiles. Pyroptotic large EVs carried interacting protein networks of RNA-binding proteins and chromatin-associated proteins many of which are known damage-associated molecular patterns or alarmins. In contrast, we found that a subpopulation of apoptotic large EVs was characterized by the presence of dsDNA, and active caspase-3/7. Together, our data shed light on the specific protein cargo of large EVs released by cells during apoptosis and pyroptosis. This study identifies candidate markers of large EVs released by dying cells and may enhance our understanding of the role of EVs in regulated cell death.

## 1. Introduction

Extracellular vesicles (EVs) are lipid bilayer-enclosed particles secreted by all cells into their surrounding environment. EVs are not only key players of cellular homeostasis, but they are also critical mediators of intercellular communication transferring proteins, lipids, and nucleic acids to recipient cells and thereby influencing several physiological and pathological processes [1]. They transport bioactive internal and external cargos that reflect both the physiological or pathological states of the cells of origin and their environment. EVs have central roles in immune regulation, angiogenesis, cancer progression, and inflammation. EVs are considered as potential biomarkers for diagnostics and monitoring of diseases and responses to therapies [2]. The MISEV2023 guidelines recommend biophysical classification of EVs into the size-based categories of small EVs (sEVs, <200 nm) and large EVs (lEVs, from 200 nm up to several microns) [1,3]. Of note, most studies until now have focused mostly on sEVs rather than the large ones. This is surprising given that different EV subtypes are secreted under distinct physiological or stress conditions and may therefore carry unique functional and diagnostic information [1,4].

Since the discovery of apoptosis, several regulated forms of cell death (RCD) pathways have been identified in pathological conditions such as cancer and neurodegenerative diseases [5,6]. Distinct modalities of RCD include apoptosis, pyroptosis, necroptosis and ferroptosis. Apoptosis is a well-characterized non-inflammatory form of RCD resulting from caspase activation and characterized by cellular shrinkage and membrane blebbing. Unlike apoptosis, pyroptosis is an inflammatory type of RCD associated with the formation of gasdermin D (GSDMD) pores, cellular swelling and lysis, as well as the release of cytokines. Necroptosis is another inflammatory type of cell death with membrane disruption through activation of the RIPK3-MLKL axis. In contrast, ferroptosis is a type of inflammatory cell death, which is due to iron-dependent lipid peroxidation resulting from impaired antioxidant defenses. Recent studies provide evidence that these cell death pathways are not isolated but interconnected, with extensive crosstalk influencing disease progression and therapeutic response [7]. Similarly, the NLRP3 inflammasome has emerged as a central regulator of PANoptosis, a hybrid form of RCD integrating pyroptosis, apoptosis, and necroptosis, which has been implicated in the pathogenesis of multiple inflammatory diseases [8]. These findings highlight that cell death is a highly dynamic and integrated process with broad implications for both health and disease [7].

Among the different RCD modalities, apoptosis and pyroptosis are of special interest due to their contrasting biological consequences and emerging interconnections. Apoptosis, classically mediated by executioner caspases such as caspase-3 and -7, dismantles cellular structures while preserving membrane integrity until late stages. This form of cell death contributes to tissue homeostasis and the clearance of damaged or infected cells without provoking strong immune responses. In contrast, pyroptosis is a lytic process triggered by inflammasome activation [9]. Canonical pyroptosis involves caspase-1–mediated cleavage of GSDMD, whereas non-canonical pathways rely on caspase-4/5 (humans) or caspase-11 (mice). Activated gasdermin forms membrane pores, leading to cell lysis and the release of inflammatory mediators such as IL-1β and IL-18. While apoptosis has a key role in development and tissue turnover, pyroptosis is essential in pathogen clearance. Excessive pyroptosis contributes to immune dysregulation and organ dysfunction [9].

An increasing body of evidence suggests that EVs are not passive byproducts of the cell death processes, but active mediators of immune regulation and disease progression. EVs produced during apoptosis have been shown to modulate immune responses in the context of autoimmunity, infection, and cancer, highlighting their diagnostic and therapeutic potential [10,11]. Even though most published data are based on studies of small apoptotic EVs, there are evidences clearly proving the immunomodulatory effect of large apoptotic EVs (ApoBDs) as well [12,13].

More recently, ApoBDs have been shown to be promising EV-based therapeutic tools, although their translational application remains poorly explored [14]. Beyond apoptosis-derived EVs, there is an increased interest in EVs generated by cells undergoing pyroptosis. Engineered versions of pyroptotic EVs have been shown to function as potent personalized cancer vaccines, outperforming other tested EV types in preventing tumor recurrence [15]. Similarly, in vascular biology, pyroptotic EVs released from endothelial cells have been shown to cause exacerbation of inflammation and lead to dysfunction, suggesting that they can act as amplifiers of pathology in atherosclerosis [16]. These findings underscore the dual roles of apoptotic and pyroptotic EVs as both therapeutic opportunities and pathological mediators.

Still, the molecular composition and heterogeneity of lEVs derived from apoptotic or pyroptotic cells remain to be further characterized to improve their use as RCD biomarkers [17]. This study was undertaken to further characterize lEVs generated during apoptosis and pyroptosis, using human monocytic models. By directly comparing the molecular signatures of apoptotic and pyroptotic vesicles, we found molecular markers associated with lEVs released by either apoptosis or pyroptosis or by both types of cell death.

## 2. Results

### 2.1. Induction of Apoptosis and Pyroptosis in U937 and THP-1 Cells

**Figure 1:**
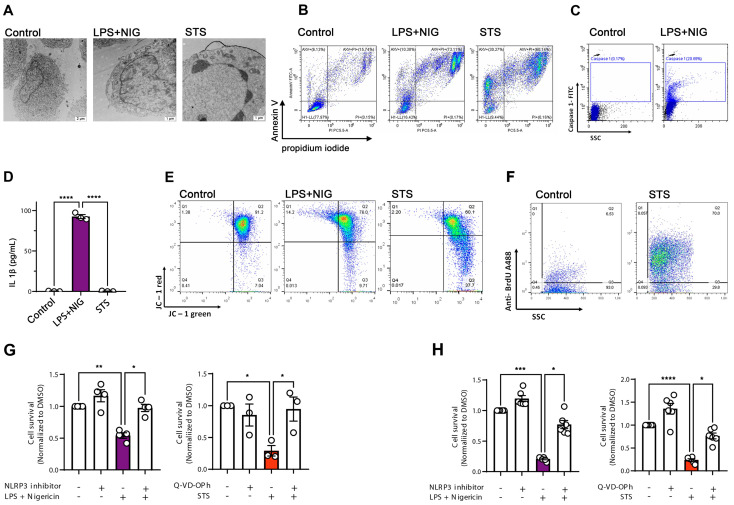
Induction of apoptosis and pyroptosis in monocyte cell lines. (**A**) Representative transmission electron microscopy (TEM) images of U937 cells under control conditions, after LPS + nigericin (NIG) treatment, and after staurosporine (STS) treatment. (**B**) Flow cytometry analysis of phosphatidylserine exposure and plasma membrane integrity by Annexin V/propidium iodide (PI) staining in THP-1 cells treated with LPS+NIG or STS. (**C**) Detection of active caspase-1 by FLICA staining in U937 cells under control and LPS+NIG treatment conditions. (**D**) Quantification of IL-1β release in cell supernatants after LPS+NIG and STS treatment, measured by ELISA. (**E**) Analysis of mitochondrial membrane potential (ΔΨm) using JC-1 dye in control, LPS+NIG, and STS-treated THP-1 cells. (**F**) Assessment of apoptosis by TUNEL assay showing DNA fragmentation in control and STS-treated cells. (**G**) Cell viability measured by CellTiter-Glo assay, based on ATP levels, in U937 cells after LPS+NIG and STS treatment. (**H**) Cell viability measured by CellTiter-Glo assay in THP-1 cells after LPS+NIG and STS treatment. Data are represented as mean ± SEM (n = 3–6 independent experiments). * *p* < 0.05, ** *p* < 0.01, *** *p* < 0.001, **** *p* < 0.0001 (one-way ANOVA with post hoc test). Scale bars for control 2 µm and STS and LPS+NIG: 1 µm.

We investigated the U937 and THP-1 monocytic cell lines, known to be sensitive to both apoptotic and pyroptotic stimuli [18,19,20]. To induce an NLRP3-inflammasome-dependent pyroptosis, lipopolysaccharide (LPS) was used to prime cells, followed by exposure to nigericin (NIG). Because staurosporine (STS) is typically identified as an apoptosis inducer, we exposed cells to STS, and then we compared the ultrastructure of the STS-challenged cells with cells treated with DMSO and LPS+NIG (Figure 1A). Analysis of U937 cells using transmission electron microscopy showed that both LPS+NIG and STS led to cellular damage; however, with distinctive features. Although cells challenged by LPS+NIG displayed an intact nucleus, they showed the presence of vacuoles and several cytoplasmic protrusions. These ultrastructural features are consistent with those reported for microglial cells undergoing pyroptosis [21]. In contrast, STS-treated apoptotic cells were shrunken, some of them contained fragmented nuclei (Figure 1A). To assess cellular viability after STS and LPS+NIG treatments, cells were analyzed by flow cytometry using Annexin V and propidium iodide (PI) labeling (Figure 1B). Compared to the DMSO-treated (control) cells, LPS+NIG exposure resulted in over 70% of Annexin V/PI double-positive pyroptotic cells. On the other hand, there was a slightly lower increase in Annexin V/PI double positivity in STS-treated late apoptotic cells (~60%), together with an increase in Annexin-V single-positive cells (30%). Pyroptosis is associated with caspase-1 (CASP-1) activation and the induction of IL1β release [22]. To confirm the activation of pyroptosis in cells treated with LPS+NIG, THP-1 monocytes were labeled with CASP-1-specific antibodies, and then analyzed by flow cytometry. Indeed, there was an increase in the percentage of CASP-1 positivity in cells treated with LPS+NIG (Figure 1C), which was also associated with a dramatic increase in the concentration of IL1β in the conditional medium of the cells (Figure 1D). Loss of mitochondrial membrane potential (∆ψm) is often associated with apoptosis [23]. Using a mitochondrial ∆ψm-sensitive dye, JC-1, to detect the mitochondrial membrane potential, we found that STS-treated cells displayed a dissolution of J-aggregates into monomers, leading to a shift from red to green fluorescence (Figure 1E). Furthermore, in cells challenged with STS, we detected DNA fragments by the APO-BrdU TUNEL Assay (Figure 1F). Together, these data suggest that STS and LPS+NIG induce typical apoptotic and pyroptotic features, respectively. We also performed cell death assays in which cell survival was quantified in the presence and absence of cell death inhibitors in U937 and THP-1 cells (Figure 1G and H, respectively). MCC950, a specific NLRP3 inhibitor was used to prevent LPS+NIG-induced pyroptosis, and Q-VD-OPh, a pan-caspase inhibitor, was used to inhibit STS-induced apoptosis [24]. Cell survival was monitored using the Cell Titer-Glo kit, as previously described [25]. LPS+NIG significantly reduced cell viability both in U937 cells, and in THP-1 cells. The presence of the NLRP3 inhibitor improved cell survival in both cell lines, confirming that LPS+NIG triggers a NLRP3-dependent pyroptosis. Similarly, STS treatment decreased cell survival and this effect was prevented in the presence of Q-VD-OPh. Our data, therefore, confirm that STS elicits apoptosis in our cells. Apoptosis and pyroptosis were also induced in HeLa cells to test if our findings can be further extended (Supplementary Figure S4).

### 2.2. EVs Released by Apoptosis and Pyroptosis

**Figure 2:**
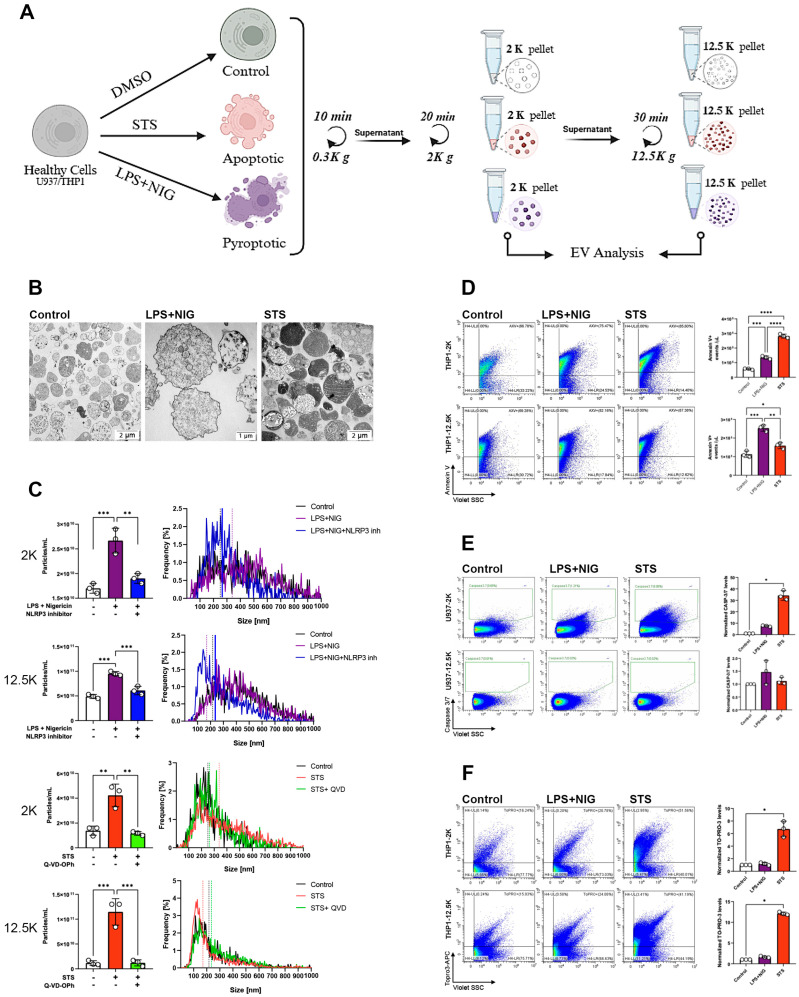
Characterization of extracellular vesicles released from apoptotic and pyroptotic monocytes. (**A**) Experimental workflow: THP−1 cells were treated with DMSO (control), staurosporine (STS; apoptotic stimulus), or LPS + nigericin (LPS+NIG; pyroptotic stimulus). Conditioned media were subjected to sequential centrifugation to isolate EV fractions at 2000× *g* (2K pellet) and 12,500× *g* (12.5K pellet) for downstream analyses. (**B**) Transmission electron microscopy (TEM) images of vesicles isolated from control, LPS+NIG−, and STS−treated U937 cells. (**C**) Quantification of EV release (left) and size distribution (right) from control, LPS+NIG-, and STS-treated cells in 2K and 12.5K fractions. (**D**) Representative flow cytometry profiles of Annexin V^+^ EVs in 2K and 12.5K fractions from control, LPS+NIG−, and STS-treated cells. (**E**) Flow cytometry analysis of EV−associated active caspase−3/7. (**F**) Flow cytometry analysis of EV membrane integrity using TO-PRO-3 in vesicles isolated from control, LPS+NIG−, and STS-treated cells. Data are presented as mean ± SEM (n = 3). * *p* < 0.05, ** *p* < 0.01, *** *p* < 0.001, **** *p* < 0.0001 (one−way ANOVA with post hoc test).

Having validated our experimental protocols to induce either apoptosis or pyroptosis in monocytes (by stimulation with STS and LPS+NIG, respectively), we sought to separate and characterize the EVs that were specifically released during the activation of the two distinct RCD pathways. Because EV size is a central biophysical parameter of EV characterization, two lEV subpopulations were investigated in parallel: lEVs separated by 2000× *g* (2K) and those isolated by 12,500× *g* (12.5K) centrifugations (Figure 2A). To validate the vesicular nature of the preparations, isolated 2K pellets derived from DMSO-treated (control), apoptotic (STS) and pyroptotic (LPS+NIG) cells were first analyzed by transmission electron microscopy. They displayed features that resemble the typical ultrastructure of lEVs (Figure 2B). However, while pyroptotic lEVs had a relatively loose and, moderately electron-dense inner structure, STS-induced apoptotic lEVs (ApoBDs) included large vesicles with highly heterogeneous electron density. We hypothesize that the very high electron density of some of the ApoBDs was due to the presence of fragmented chromatin in ApoBDs. The lEVs were then analyzed by nanoparticle tracking analysis (NTA) to examine the concentration of the particles released by cells during apoptosis and pyroptosis with or without apoptotic/pyroptotic inhibitors (Figure 2C). Both LPS+NIG- and STS-treated cells showed a significant increase in lEV release, regardless of the isolation protocol. In all conditions, the NLRP3 inhibitor alleviated the induction of EV release by pyroptotic cells, and the pan-caspase inhibitor Q-VD-OPh alleviated the EV release by apoptotic cells (Figure 2C). We further validated these findings using the Micro Bicinchoninic Acid (BCA) protein assay and the sulfo-phospho-vanillin (SPV) lipid assay (Supplementary Figures S1A and S1B, respectively). Both assays demonstrated a marked increase in EV release following induction of cell death compared with control conditions or treatments with the corresponding inhibitors. These data suggest that both apoptotic and pyroptotic types of cell death are characterized by an increase in EV release. Annexin-V binding to apoptotic and pyroptotic EVs was analyzed by flow cytometry (Figure 2D). In the case of THP1-derived lEVs, both STS and LPS+NIG stimulation significantly increased the proportion of Annexin-V-positive 2K and 12.5K vesicles. The gating strategy is shown in Supplementary Figure S2. Beyond Annexin-V binding to the surface of EVs, increased caspase 3/7 expression is often associated with apoptosis induction in cells [26]. To determine if the apoptotic caspase activity was detectable in EVs induced during the two investigated cell death pathways, we used a fluorogenic substrate of caspase 3/7 (Figure 2E). No significant increase in the active caspase 3/7 activity was found in lEVs derived from LPS+NIG treated pyroptotic cells. In contrast, in STS-induced 2K EVs, a significant increase in caspase-3/7 activity was observed, which was not detected in the 12.5K pellet (Figure 2E). Finally, we investigated the presence of dsDNA in lEVs, using TO-PRO-3 labeling. Apoptosis is usually associated with an active degradation of the chromatin and the formation of large ApoBDs carrying nucleus-derived content. Only apoptosis-derived lEVs showed an increase in the TO-PRO-3 labelling (7-fold increase in 2K, and 13-fold increase in 12.5K EVs compared with the controls, while no increase was observed in the pyroptosis-derived EVs) (Figure 2F). Thus, our data suggest that the presence of dsDNA is characteristic of apoptotic lEVs.

### 2.3. Differential Expression of the Main Tetraspanins of Apoptotic and Pyroptotic lEVs

**Figure 3:**
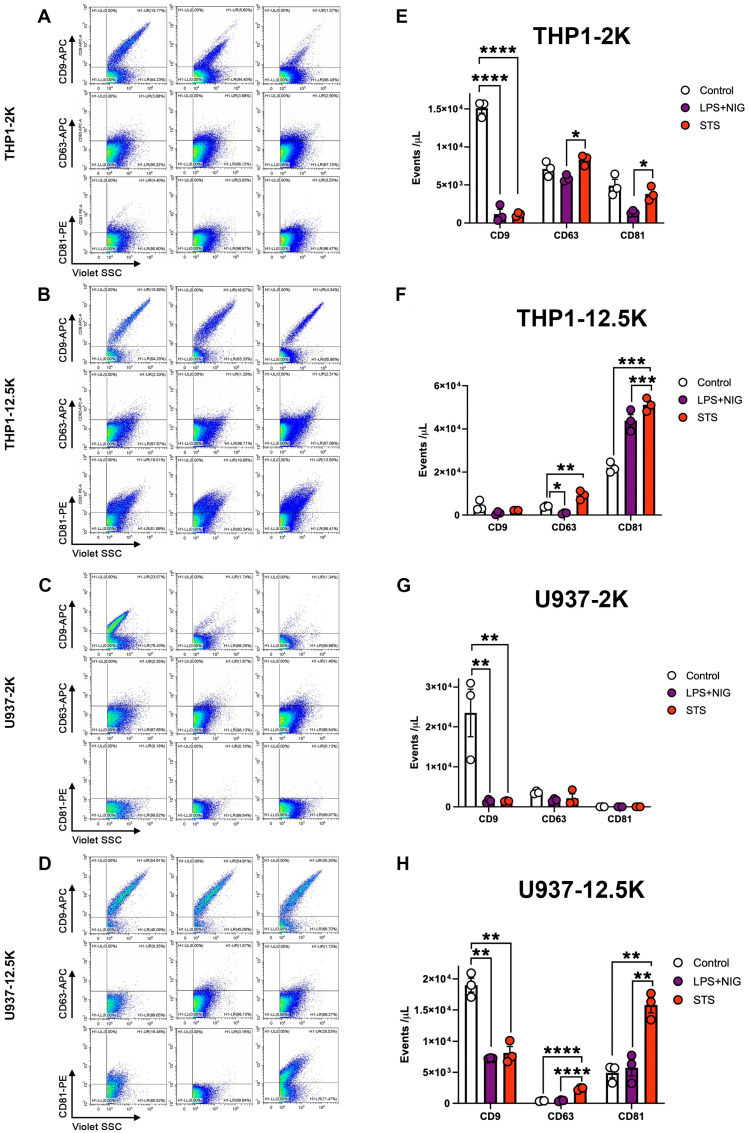
Surface marker characterization of extracellular vesicles (EVs) released from apoptotic and pyroptotic monocytes. Representative flow cytometry plots and quantification of EV surface markers CD9^+^, CD63^+^, and CD81^+^ in vesicles isolated from control, LPS + nigericin (LPS+NIG)-, and staurosporine (STS)-treated cells. (**A**,**E**): THP-1-derived EVs (2K pellet), and (**B**,**F**): THP-1-derived EVs (12.5K pellet), (**C**,**G**): U937-derived EVs (2K pellet), (**D**,**H**): U937-derived EVs (12.5K pellet). Data are presented as mean ± SEM (n = 3 independent experiments). * *p* < 0.05, ** *p* < 0.01, *** *p* < 0.001, **** *p* < 0.0001 (one-way ANOVA with post hoc test).

EV membranes are enriched in tetraspanin proteins such as CD9, CD63, and CD81 and these proteins are considered as EV markers [27]. To investigate how apoptosis and pyroptosis may affect the molecular composition of released lEVs, we next assessed the tetraspanin positivity of lEVs by flow cytometry in the case of both U937 and THP-1-derived vesicles (Figure 3). Both apoptotic and pyroptotic cell-derived lEVs, separated by a 2K centrifugation, displayed a striking reduction in CD9 expression compared to control cell-derived EVs. The 12.5K lEVs released by U937 and THP-1 cells showed a significantly increased CD63 expression after apoptosis induction. In addition, in 12.5K lEVs, STS treatment induced a consistent increase in the expression of CD81 on lEVs regardless of the cell type. LPS+NIG treatment, on the other hand, modified the expression of CD81 only on the surface of THP1 12.5K EVs (Figure 3). Taken together, our data suggest that tetraspanin proteins are differentially expressed on EVs derived from apoptotic and pyroptotic cells. However, the tetraspanin expression patterns of EVs do not mirror the expression on the surface of the releasing cells (Supplementary Figure S8).

### 2.4. Analysis of the Protein Composition of Pyroptotic and Apoptotic EVs

**Figure 4:**
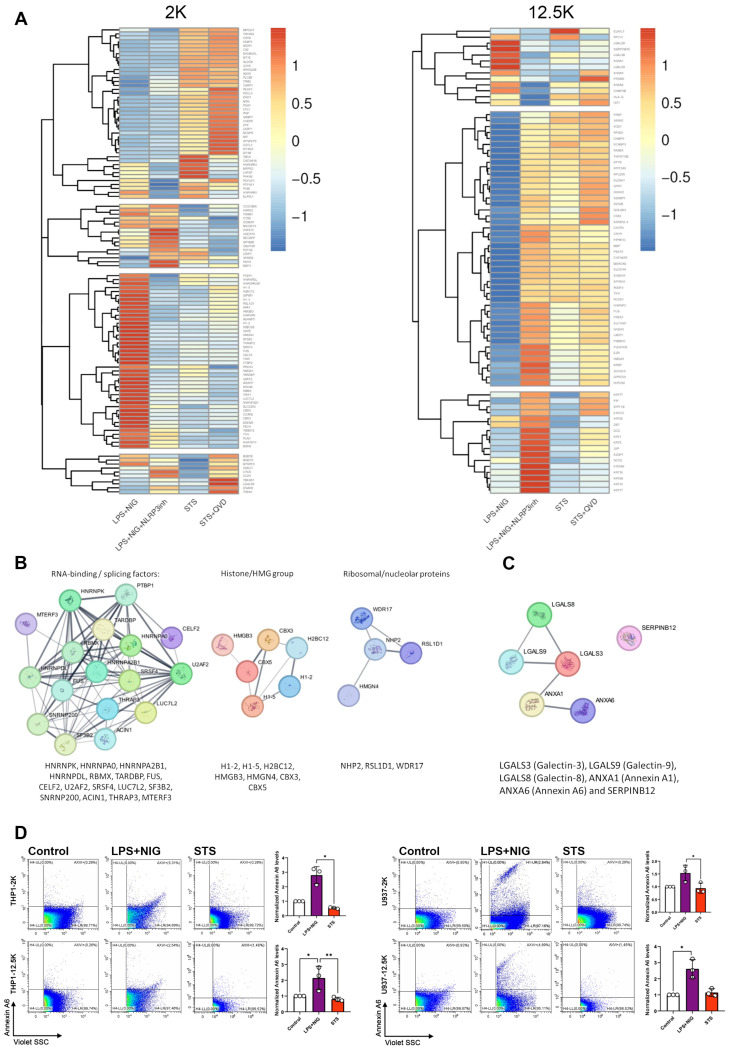
Proteomic profiling and validation of Annexin A6 expression. (**A**) Heatmaps showing hierarchical clustering of differentially expressed proteins identified by mass spectrometry across experimental groups LPS+NIG/LPS+NIG+NLRP3inh/STS/ STS+QVD. Protein expression values were normalized and are represented by Z-scores (red = high, blue = low). Protein–protein interaction (PPI) networks generated using STRING [28,29] depicting clusters of proteins in LPS+NIG in 2K and (**B**) 12.5K pellets (**C**). (**D**) Flow cytometry analysis of Annexin A6 expression in U937 and THP1-derived EVs: representative scatter plots (left) and quantification of Annexin A6–positive EVs (right). Data are presented as mean ± SEM (n = 3 independent experiments) (* *p* < 0.05, ** *p* < 0.01).

Finally, we sought to analyze the protein content, which characterizes apoptotic and pyroptotic EVs. Pyroptosis of THP1 cells was induced in the presence and absence of an NLRP3 inhibitor, and apoptosis was induced with or without the inhibitor Q-VD-OPh (QVD). EVs were harvested and analyzed by mass spectrometry. The lEV-associated protein patterns are illustrated by the heatmap (Figure 4A). Proteins enriched in the 2K and 12.5K pellets (control, LPS+NIG, LPS+NIG with NLRP3 inhibition, STS and STS combined with Q-VD-OPh) were analyzed. Proteins, the abundance of which showed significant change compared with the control, are listed in Supplementary Table S2. Figure 4B indicates a few protein–protein interaction (PPI) networks, which were identified in the pyroptotic 2K EVs. Hub proteins in these networks belonged to the RNA-binding/splicing factors (HNRNPK, HNRNPA0, HNRNPA2B1, HNRNPDL, RBMX, TARDBP, FUS, CELF2, U2AF2, SRSF4, LUC7L2, SF3B2, SNRNP200, ACIN1, THRAP3, MTERF3), (Figure 4B), chromatin-associated proteins (e.g., H1-2, H1-5, H2BC12, HMGB3, HMGN4, CBC3, CBX5) and ribosomal/nucleolar proteins (such as NHP2, RSL1D1, WDR17) (Figure 4B). The 12.5K pyroptotic EVs included a small network of galectin proteins (LGALS3, LGALS8 and LGALS9), annexins (ANXA1 and ANXA6) and SERPINB12 (Figure 4C). Interestingly, ANXA6 was more abundant in LPS+NIG-induced lEVs than those induced by STS (Figure 4D), suggesting that ANXA6 is more characteristic for pyroptotic EVs than the apoptotic ones. ANXA6 expression was also tested on HeLa cell-derived EVs and similarly to what we found in the case of THP1 and U937 cells, we observed an increased expression upon induction of pyroptosis (Supplementary Figure S5). Of note, we also detected the up-regulation of ANXA6 in the presence of Q-VD, but independently of cell death execution. Taken together, our mass spectrometry data suggest that pyroptotic EVs are characterized by the expression of proteins, which can differ between the two EV size-based populations we investigated.

### 2.5. Analysis of the Intercellular Spread of Cell Death Signals by Pyroptotic or Apoptotic EVs

We exposed naïve THP1 and U937 cells to EVs released by apoptotic and pyroptotic cells. We found that the 2K apoptotic EVs of THP1 cells increased the ratio of Annexin V/PI positive cells after 24 h incubation. Similarly, we detected significant increase in the ratio of Annexin V/PI positive U934 cells upon 24 h exposure to both apoptotic and pyroptotic cell-derived 2K EVs and apoptotic 12.5K EVs. These data provide evidence of the transmission of RCD-related signals among cells by lEVs (Supplementary Figure S6). To investigate whether the pyroptotic phenotype (characterized by IL-1β secretion) could be horizontally transferred via lEVs, we separated lEVs from the conditioned media of cells exposed to various cell death inducing stimuli and incubated them with recipient U937 and THP-1 monocytic cells at a ratio of 10,000 EVs/cell. Following 24 h incubation, we measured the levels of IL-1β in the conditioned medium using an ELISA. In the case of THP1-derived 2K lEVs, we observed a significant increase in IL1β secretion, when the EV releasing cells had been previously exposed to LPS+NIG-EVs (*p* < 0.01, ANOVA). A similar trend was observed in U937 cells, where LPS+NIG-EVs induced the highest levels of IL1β secretion compared to all other groups (Supplementary Figure S7). These data indicate that EVs produced during pyroptosis carry bioactive cargo capable of triggering an inflammatory response in healthy recipient cells (thus, effectively “spreading” the pyroptotic signal).

## 3. Discussion

The field of EVs has expanded exponentially in recent years, and numerous disease-related EV molecules have been suggested as candidate biomarkers. However, limited attention has been paid to EVs released during regulated cell death (RCD) processes, in particular to the large ones (lEVs). While it would be important to distinguish EVs released by healthy cells and those discharged by dying cells in liquid biopsy samples, we still do not have molecular markers for such a distinction. Importantly, even fundamental questions remain to be answered, such as whether EVs with externalized phosphatidyl serine (i.e., Annexin V-positive EVs) are exclusively derived from apoptotic cells [30] or whether they can also originate from donor cells upon activation [31].

To address this scientific gap, our current study was designed to investigate EVs generated by cells undergoing apoptosis or pyroptosis under well-controlled conditions. We selectively induced apoptosis or pyroptosis and both pathways were pharmacologically validated using inhibitors (Q-VD-OPh for apoptosis, MCC950 for pyroptosis), ensuring that the observed cell death belonged to the appropriate RCD pathway.

The use of RCD inhibitors inevitably introduces certain limitations, especially for caspase-dependent cell death pathways. Indeed, apoptosis and pyroptosis are complex and highly interconnected processes that require a large number of distinct proteins that are not exclusively involved in the execution ofRCD. Apoptosis can proceed through intrinsic or extrinsic pathways, involving distinct caspase proteases and mitochondrial proteins. Therefore, potent apoptosis inhibition requires pan-caspase inhibitors such as Z-VAD.fmk or Q-VD-OPh [24]. Q-VD-OPh efficiently blocks STS-elicited cell death (Figure 1G,H), similarly to Z-VAD.fmk. Q-VD-OPh also inhibits non-apoptotic caspases. In some cases, caspase inhibition can be lethal to cells by preventing the anti-necroptotic role of Caspase-8 [32,33]. Such an effect was not observed here since Q-VD-OPh treatment alone did not induce significant cell death (Figure 1G,H), suggesting that neither STS nor STS+ Q-VD-OPh triggers necroptosis. Of note, there are known roles for caspases in healthy cells [34]. This urges caution when interpreting the characterization of the STS+ Q-VAD-OPh, since this experimental condition is not only free of apoptosis execution, but also includes a deficit for caspase activity, independently of cell death pathways.

In this study, we carried out a comparative analysis of apoptotic and pyroptotic EVs derived from monocytic U937 and THP-1 cells, combining multiple EV analysis approaches (such as NTA, EM, flow cytometry and mass spectrometry) to dissect their unique characteristics. Apoptosis and pyroptosis are both immunologically relevant RCD pathways with profound implications in health and disease. While apoptosis is traditionally regarded as a non-inflammatory and homeostatic form of cell death [35], it has been increasingly recognized that failure of apoptotic cell clearance can lead to inflammation and autoimmunity [36]. Conversely, pyroptosis is overtly pro-inflammatory and plays a central role in the control of infection, chronic inflammatory disorders, and sepsis [37]. The significance of distinguishing apoptotic and pyroptotic EVs extends beyond biomarker discovery. Both types of vesicles may actively modulate intercellular communication, inflammation, and tissue regeneration. For instance, stem cell-derived apoptotic EVs have demonstrated significant regenerative potential in animal models [38], suggesting that apoptotic EVs may actively contribute to tissue repair.

By analyzing monocyte-derived apoptotic and pyroptotic lEVs, we found that the release of lEVs was markedly increased under both cell death conditions, reinforcing the notion that cell death programs actively shape vesiculation. The 2K apoptotic pellet of lEVs revealed distinct molecular cargo, including apoptotic markers such as caspase-3/7 activity and dsDNA (Figure 2E,F), and the pyroptotic lEVs also included differentially expressed proteins such as Annexin A6 (Figure 4D). The 12.5K pyroptotic EVs showed a markedly increased expression of CD81 upon pyroptosis induction, and a network of RNA-binding proteins was found in pyroptotic cargos (Figure 4B). These findings underscore the urgent need to reconsider the role of lEVs in liquid biopsy strategies, particularly because they may carry the most direct signatures of RCD execution. This point is critical because current EV research, especially in clinical contexts, often excludes lEVs when enriching for sEVs. Our data strongly suggest that such an exclusion may result in the loss of a substantial portion of RCD-specific information, which might be relevant for diagnostics or therapeutic monitoring. A key challenge in the field lies in the accurate identification of RCD -derived EVs within complex biological fluids. While Annexin V positivity is widely used to characterize apoptotic cells and EVs, our results clearly show that pyroptotic EVs also bind Annexin V. This highlights that testing Annexin V binding alone cannot discriminate between EVs derived from cells undergoing apoptosis and pyroptosis. Likewise, expression of classical EV markers such as CD9, CD63, and CD81 are modulated in a context-dependent manner but are not uniquely specific to either cell death pathway. These findings underscore the necessity of developing combinatorial marker strategies to reliably determine EV origin. Previous work has already suggested potential RCD-specific EV markers, such as Plexin B2, which was shown to be a regulator of monocyte apoptotic cell disassembly [39]. In our MS analysis, we also identified Plexin B2 in the 2K and 12.5 large EV pellets; however, we could not detect a significant difference in its abundance in apoptotic and pyroptotic lEVs.

In pyroptotic lEVs, we identified a network of interacting RNA-binding proteins involved in pre-mRNA splicing and broader RNA metabolism (splicing, stability, export, translation, stress granules). This protein signature was abrogated by NLRP3 inhibition, supporting its mechanistic link to inflammasome-driven death (Supplementary Figure S3). Pyroptotic lEVs carried (i) a protein network of chromatin-associated proteins involved in regulating chromatin structure and gene expression, (ii) a network of nucleolar/nucleus-enriched proteins linked to ribosome biogenesis and RNA metabolism. Importantly, several of the above proteins such as histones (H1-2, H1-5, H2BC12), HMGB3 (High mobility group box 3) and HMGN4 are known DAMPs [40]. Galectins, including LGALS3, LGALS8, and LGALS9 can act as alarmins [41], promoting immune activation. These findings are consistent with the pro-inflammatory nature of pyroptotic cell death. The enrichment of these proteins in pyroptotic lEVs raises questions about their potential role in recipient cells. Our data presented here confirms that pyroptotic lEVs may amplify immune responses during infection or sterile inflammation by horizontally transferring pro-inflammatory factors. As the inflammatory status and the kinetics of resolution are central to tissue healing, RCD-derived EVs should be further investigated as functional mediators of pathophysiological processes. Our findings highlight candidate EV markers while also pointing out some challenges for the field. First, identification of robust markers for apoptotic versus pyroptotic EVs will likely require combinatorial approaches that integrate multiple markers rather than relying on single proteins or biophysical traits. Second, the current limited consideration of large EVs in both experimental and clinical workflows should be reevaluated, as these EV populations appear to harbor unique and informative molecular cargo. Third, understanding the biological roles of RCD-derived EVs may be critical for harnessing their diagnostic and therapeutic potential. Taken together, this study provides novel data for distinguishing apoptotic from pyroptotic EVs at the structural, biochemical, and proteomic levels. By identifying both shared and pathway-specific features, our data contribute to defining the molecular signatures necessary for the next generation of lEV-based liquid biopsies and for elucidating the intercellular functions of death-associated vesicles.

## 4. Materials and Methods

### 4.1. Reagents

Information about all reagents used in this study is listed in Supplementary Table S1.

### 4.2. Cell Lines and Cell Death Induction

THP-1 cells (human monocytes derived from the peripheral blood of a male with acute monocytic leukemia) and U937 cells (pro-monocytic cells derived from the pleural effusion of a male with histiocytic lymphoma) and HeLa cells (human cervical adenocarcinoma cells) were used in this study and have been purchased from ATCC. THP1 and U937 cells were cultured in RPMI-1640 medium (Gibco, Thermo Fischer, Waltham, MA, USA) and HeLa cells were grown in DMEM (Gibco) supplemented with 10% fetal bovine serum (FBS; BioSera, Cholet, France). Cells were additionally supplemented with 1% penicillin–streptomycin (Sigma-Ald, St. Louis, MO, USA), 1% L-glutamine (Sigma-Aldrich), and 0.1% HEPES (Sigma-Aldrich). THP-1 cells were cultured at densities of 2 × 10^5^–1 × 10^6^ cells/mL, whereas U937 cells were cultured at 1 × 10^5^–2 × 10^6^ cells/mL. All cultures were incubated at 37 °C in a humidified atmosphere containing 5% CO_2_. Apoptosis was induced by treatment with staurosporine (STS; Sigma-Aldrich, 2.5 μM, 4 h). For inhibition experiments, the pan-caspase inhibitor Q-VD-OPh (Sigma-Aldrich) was added 2 h prior to STS exposure. Pyroptosis was induced by sequential stimulation with lipopolysaccharide (LPS; Sigma-Aldrich, 2 μM, 3 h) followed without washing by nigericin (NIG; InvivoGen, San Diego, CA, USA, 10 μM, 1 h). For inhibition experiments, cells were pre-treated with the NLRP3 inhibitor (Sigma-Aldrich, 10 μM) 1 h before LPS+NIG stimulation.

### 4.3. Cell Viability Assays

Each cell line was incubated for 24 h prior to STS treatment. Cell viability was assessed 4 h after STS induction using the CellTiter-Glo^®^ 2.0 Assay (Promega, Budapest, Hungary). This luminescent assay measures cell viability by quantifying the adenosine triphosphate (ATP) present in metabolically active cells. ATP levels are directly proportional to the number of viable cells, and luminescence was recorded as relative light units (RLU) using LS-50B Spectrometer (PerkinElmer Instruments, Shelton, CT, USA).

### 4.4. Electron Microscopy (EM) Imaging

Cells and EV pellets were fixed in 4% paraformaldehyde (PFA) in PBS for 60 min at room temperature (Sigma-Aldrich). Following fixation, the samples were washed in PBS and post-fixed with 1% osmium tetroxide (OsO_4_; Taab, Aldermaston, UK) for 15 min, rinsed with distilled water, and dehydrated in a graded ethanol series. Block staining was carried out with 1% uranyl acetate in 50% ethanol for 30 min. Samples were embedded in Taab 812 resin (Taab) and polymerized overnight at 60 °C. Ultrathin sections (50–70 nm) were obtained using a Leica UCT ultramicrotome (Leica Microsystems, Wetzlar, Germany). Sections were examined with a Hitachi 7100 transmission electron microscope (Hitachi Ltd., Tokyo, Japan) equipped with a Veleta 2k × 2k side-mounted TEM CCD camera (Olympus, Tokyo, Japan). Some of the EN images have been produced using a FEI Tecnai 12 transmission electron microscope (Fei). Contrast and brightness of electron micrographs were adjusted by Adobe Photoshop CS3 CC 2026 (Adobe Photoshop Incorporation, San Jose, CA, USA).

### 4.5. TUNEL Assay

U937 Cells were treated with 2.5 µM STS for 4 h, while untreated cells served as controls. Following incubation, cells were washed, fixed in 1% paraformaldehyde for 15 min on ice, and permeabilized in 0.1% Triton X-100/0.1% sodium citrate (ice-cold) for 2 min before washing in PBS. DNA strand breaks were labeled using the APO-BrdU TUNEL Assay Kit with Alexa Fluor™ 488 Anti-BrdU (ThermoFisher, Waltham, MA, USA 23210) according to the manufacturer’s instructions. Briefly, fragmented DNA ends were enzymatically labeled with 5-bromo-2’-deoxyuridine 5’-triphosphate (BrdUTP) by terminal deoxynucleotidyl transferase (TdT), and BrdU incorporation was detected using an Alexa Fluor 488–conjugated anti-BrdU antibody. To assess total DNA content, cells were counterstained with propidium iodide (PI; 50 µg/mL) for 15 min at room temperature and were protected from light. Samples were acquired on a FACS Calibur Flow Cytometer using 488 nm excitation, with Alexa Fluor 488 detected in the FL1 channel and PI in FL3/FL2.

### 4.6. MitoProbe™ JC-1 Assay

Mitochondrial membrane potential in U937 cells was evaluated using the JC-1 dye (Thermo Fisher). Following treatment with STS or LPS+NIG, cells were resuspended at 1 × 10^6^ cells/mL in PBS. For positive controls, cells were treated with 50 μM CCCP for 5 min at 37 °C. JC-1 was then added to a final concentration of 2 μM, and cells were incubated for 15–30 min at 37 °C in 5% CO_2_. For additional labeling, cells were incubated with Annexin V–allophycocyanin in annexin binding buffer (10 mM HEPES, 140 mM NaCl, 2.5 mM CaCl_2_, pH 7.4) for 15 min at 37 °C. After washing, cells were resuspended in PBS or binding buffer and analyzed by flow cytometry by a fluorescent emission shift from green (~529 nm) to red (~590 nm) (ThermoFisher).

### 4.7. Measurement of Cytokine Concentrations by ELISA

IL-1β levels were measured in the culture supernatants using an IL-1β ELISA kit (Sigma-Ald, St. Louis, MO, USA, RAB0273). Supernatants were processed and analyzed according to the manufacturer’s instructions. Briefly, standards and samples were added to antibody-coated 96-well plates, incubated, washed, and then incubated with a detection antibody and TMB substrate. The reaction was stopped, and absorbance was measured at 450 nm using a microplate reader (Biosan Microplate Photometer HiPo MPP-96). IL-1β concentrations were calculated from a standard curve and compared between control and treated groups.

### 4.8. Extracellular Vesicle Separation

Cells were cultured under serum-free conditions for 4 h prior to induction of cell death. EVs were isolated using a combination of multistep differential centrifugation and hydrostatic filtration. Hydrostatic filtration was applied to preserve EV integrity and to minimize fragmentation typically caused by high-pressure filtration. To distinguish vesicle populations, fractions obtained after centrifugation at 2000× *g* were designated as large EVs (lEVs, 2K pellet), and those obtained after 12,500× *g* were designated as 12.5K lEVs. An Eppendorf centrifuge 5804R was used for pelleting cells and 2K lEVs, an Avanti JX26 centrifuge (rotor: JA 25.15) was used for pelleting 12.5K EVs, and Eppendorf 5424 R (rotor FA-45-24-11) was used for washing the lEVs. Cells were collected from culture flasks and transferred into 50 mL tubes. Cells were removed by centrifugation at 300× *g* for 5 min (Beckman Coulter, Brea, CA, USA). This step was repeated twice to ensure the complete removal of intact cells. The resulting supernatant was filtered by gravity through a 5 μm pore-size filter (Merck Millipore, Darmstadt, Germany) pre-equilibrated with PBS. The filtrate was centrifuged at 2000× *g* for 20 min at 4 °C, and the pellet was washed with 1000 μL PBS by centrifugation at 2000× *g* for 20 min at 4 °C in 1.5 mL microcentrifuge tubes (Eppendorf, Hamburg, Germany). The resulting supernatant was filtered through a 0.8 μm pore-size Whatman CA filter (Sigma-Ald, St. Louis, MO, USA) and centrifuged at 12,500× *g* for 30 min at 4 °C (Avanti JX-26, rotor JA-25.15, Beckman Coulter). The pellet was washed with 1000 μL PBS and centrifuged again at 12,500× *g* for 30 min at 4 °C in an (Eppendorf, Hamburg, Germany). centrifuge. Finally, both the 2K and 12.5K pellets were resuspended in the appropriate buffer for downstream experiments.

### 4.9. Determination of Size Distribution and Concentration of EVs

EVs were characterized by nanoparticle tracking analysis (NTA). For NTA, 2K and 12.5K EV pellets were examined using a ZetaView Z-NTA system (S/N: 19-459 ZetaView Particle Tracking Analyzer PMX-120 instrument, Particle Metrix, Munich, Germany). Each sample was measured across 11 positions in two cycles to ensure reproducibility. Measurements were conducted at 25 °C to maintain consistent Brownian motion, and reliable detection was achieved at concentrations of 150–300 particles per frame. For 2K lEVs, parameters were set to a maximum area of 1000, a minimum area of 5, and a minimum particle brightness of 20. For 12.5K LEVs, the camera settings were adjusted to sensitivity 80 and shutter speed 100, with analysis parameters of maximum area 200, minimum area 10, and minimum particle brightness 30. Data were processed using ZetaView software (v.8.05.11 SP2).

### 4.10. Total Protein and Lipid Concentration Measurement

The total protein concentration of each EV preparation was determined using the Micro Bicinchoninic Acid (Micro BCA™ Protein Assay Kit Thermo Fisher Scientific, Waltham, MA, USA), following the manufacturer’s protocol. Measurements were performed with a NanoDrop (1000 Spectrophotometer Version 3.8.1 software, Thermo Fisher Scientific). Protein concentration was calculated from a standard calibration curve, and absorbance values of blank controls were subtracted from sample readings.

Lipid quantification was carried out using the sulfo-phospho-vanillin (SPV) assay. The procedure was performed on ice in Eppendorf tubes, after which the samples were transferred to a non-treated 96-well microplate (Nunc™ MicroWell™ 96-Well Microplates, 108 per box, 243656, Thermo Fisher) for incubation, following the protocol described by Visnovitz et al. [28]. For standard curve generation, liposomes composed of 1 μg/μL DOPC (1,2-dioleoyl-sn-glycero-3-phosphocholine, lyophilized powder, P6354-25MG, Sigma Aldrich) were used. To disrupt vesicles, sonication and multiple freeze–thaw cycles were applied, after which 96% sulfuric acid (339741, Sigma Aldrich) was added to 40 μL of vesicle suspension. Samples were then subjected to heat treatment at 90 °C for 20 min using an AccuBlock digital dry bath (Labnet International, Edison, NJ, USA). After cooling to room temperature, 120 μL of phospho-vanillin reagent (1 mg/mL vanillin in 17% phosphoric acid; Sigma) was added. Color development was performed in a non-treated 96-well plate (pack of 25, 266120, Thermo Fisher), with incubation for 20 min at 90 °C under shaking at 350–400 rpm. Subsequently, the plate was cooled at 4 °C for 5 min. The phospho-vanillin reagent was freshly prepared under a chemical hood by dissolving 50 mg vanillin (ReagentPlus^®^, 99%, 79617, Merck, Rahway, NJ, USA) in 50 mL of 17% phosphoric acid (85 wt.% in H2O, 99.99% trace metals basis, 345245-100ML, Merck). For each well containing standards and experimental samples, 60 μL of this reagent was added. The plate was then shaken at 350–400 rpm at 37 °C for 1 h to catalyze the color reaction. Finally, absorbance was measured at 540 nm using an ELISA reader (352 Multiskan MS LabSystems, Microplate Reader, 74165-5, Labsystem, Vantaa, Finland). The standard calibration curve was generated based on the absorbance values of DOPC standards, and lipid concentrations of unknown samples were calculated accordingly.

### 4.11. Flow Cytometry

Cells, as well as 2K and 12.5K EV pellets, were analyzed using flow cytometry (FACSCalibur; BD Biosciences, San Jose, California, US (for the TUNEL assay analysis) and the rest of the experiments were carried out using a CytoFLEX S V4-B2-Y4-R3 Flow Cytometer; Beckman Coulter). Samples were stained with fluorochrome-conjugated antibodies and dyes, including Annexin V-FITC (Sony, Lot-235899), propidium iodide (PI; BD Biosciences, Franklin Lakes, NJ, USA), CD63 (Sony, Lot-195923), CD81 (Sony, Lot-158543), CD9 (Sony, Lot-162120), Annexin A6 Santa Cruz Biotechnology, Lot-F2521), TO-PRO-3, Caspase 3/7 (Invitrogen, Carlsbad, CA, USA), and Caspase 1 (Invitrogen, Carlsbad, CA, USA). Antibodies were diluted 1:10 in 0.1 μm filtered PBS and centrifuged at 15,000× *g* 1.5 min for the removal of aggregates. During Annexin V–propidium iodide staining, cells were incubated with the staining solution prepared in annexin-binding buffer (BD Biosciences). For the detection of CD63, CD81, and CD9 on EVs, samples were resuspended and stained in 0.2 µm-filtered phosphate-buffered saline (PBS). For Annexin A6 labeling, vesicle membranes were first permeabilized with 4% paraformaldehyde (PFA), followed by staining in annexin-binding buffer. Incubations were performed at 4 °C and were protected from light by covering with aluminum foil. Measurements were carried out using the “slow” flow rate (10 μL/min) for 2 min, and events were recorded within a previously optimized EV detection gate (Supplementary Figure S2). To confirm that the detected signal originated from intact vesicles, differential detergent lysis with 0.1% Triton X-100 was performed. CountCheck beads (3 μm; Sysmex Partec GmbH, Münster, Germany) were used as an internal standard to calculate the absolute particle number. Flow Cytometry Sub-micron Particle Size Reference Kit beads (Invitrogen, Lot-2104159) were employed for standardization and to establish gating strategies. A gate corresponding to the 200–1000 nm size range was defined based on Violet-SSC and FSC-H parameters using size reference beads (Supplementary Figure S2). Gating was performed on the Violet-SSC parameter, after which Annexin V–positive (or CD63, CD81, CD9), Triton-sensitive particles were classified as vesicles. Flow cytometry data were acquired and analyzed using CytExpert 2.4 software (Beckman Coulter, Brea, CA, USA), and datasets were exported to Microsoft Excel (Office 365; Microsoft, Redmond, WA, USA) for further processing according to the following formula: Absolute EV count = (Known bead count/detected bead count) × (detected positive EV events − background controls) × dilution factor.

### 4.12. Mass Spectrometry

EVs pelleted at 2K g and 12.5K g from conditioned media of THP-1 cells under five conditions (control, induction of apoptosis or pyroptosis, in the presence or absence of their respective cell death inhibitors). The samples were lysed in 0.1% Rapigest/50 mM TEAB using two freeze-sonication cycles (10 s in liquid nitrogen, 3 min sonication). Proteins were reduced with DTT (100 mM, 60 °C, 30 min), alkylated with iodoacetamide (100 mM, 30 min, RT, dark), and excess IAM quenched with DTT (10 mM). Proteins were digested with MS-grade trypsin (2–3% *w*/*w*, 37 °C, 2 h), and Rapigest was decomposed by TFA acidification and heating (10% TFA, 90 °C, 10 min). Peptides were dried, resuspended in 100 mM TEAB, and 10 μg aliquots were labeled with TMTpro 16plex reagents (1 h, RT; quenched with 1.25% hydroxylamine, 15 min). Labeled peptides were combined, diluted in 0.1% FA, and loaded onto disposable C18 tips (Evosep). Peptides were separated on an Evosep Endurance C18 column (150 μm × 15 cm, 1.9 μm) using an 88 min gradient and analyzed on an Orbitrap Lumos Fusion Tribrid MS equipped with a FAIMS Pro ion mobility device (compensation voltages (CV): −50 and −70 V, in alternating 1.5 s cycles). Data were processed in Proteome Discoverer 3.0 SP1 against the SwissProt human database with trypsin specificity, carbamidomethylation as a static modification, and dynamic modifications including methionine oxidation and TMTpro labeling. Quantification used “unique+razor” peptides, normalized by total peptide amount, and background-based *t*-test assessed differential abundance. Changes in protein abundance with an adjusted *p*-value ≤ 0.05 have been considered significant, with a |log2foldchange| cutoff of 1. Three biological replicates were analyzed for each experimental group.

### 4.13. Statistical Analysis

Statistical analyses were conducted using GraphPad Prism (version 8.2.0; GraphPad Software, Boston, MA, USA). Data are expressed as mean  ±  standard deviation, with individual data points displayed where relevant. The Shapiro–Wilk test was used to assess data normality. For comparisons between two groups, normally distributed data were analyzed using an unpaired Student’s *t*-test, whereas data not showing normal distribution were analyzed using the Mann–Whitney U test. Paired data were evaluated using either a paired t-test or the Wilcoxon signed-rank test, depending on normality. For comparisons involving more than two groups, one-way ANOVA was applied, followed by Tukey’s post hoc test when appropriate; for non-parametric data, the Friedman test was used. Statistical significance was defined as *p*  <  0.05, with levels indicated as *p*  <  0.05, *p*  <  0.01, and *p*  <  0.001. The visualization of MS results was performed using R Statistical Software (v4.3.1; R Core Team 2023). The heatmaps have been generated using the heatmap package (v1.0.12). The schematic illustrations were generated with BioRender (https://www.biorender.com) and Microsoft PowerPoint (Microsoft Office Professional Plus 2016, version 16.0.4266.1001). Protein–protein interaction (PPI) networks were generated using STRING [29]. The MS data were uploaded to the MASSIVE data repository (ftp://MSV000100407@massive-ftp.ucsd.edu) accessed on 8 January 2026.

## 5. Conclusions

In this study, we characterized EVs released during apoptosis and pyroptosis by monocytic cell lines, integrating ultrastructural, biochemical, and proteomic analyses. Our data demonstrate that both forms of regulated cell death markedly enhance EV release, with lEVs carrying distinct molecular signatures that reflect the underlying death pathway. Apoptotic EVs were enriched in caspase-3/7 activity and dsDNA, whereas pyroptotic EVs carried networks of RNA-binding and chromatin-associated proteins. Importantly, we show that Annexin V binding is not exclusive to apoptotic lEVs, highlighting the need for combinatorial marker panels to reliably distinguish RCD-derived vesicles. Collectively, these findings underscore that lEVs represent an underappreciated but highly informative source of EV markers for monitoring cell death pathways. By providing comparative profiles of apoptotic and pyroptotic EVs, this work highlights the significance of lEV analysis in precision diagnostics and may offer hints for exploring their active roles in immune modulation, inflammation, and tissue regeneration.

## Data Availability

The original LC-MS raw data and Proteome Discoverer analysis result presented in the study are openly available in the ProteomeXchange repository (https://www.proteomexchange.org/), identifier: PXD072811.

## Supplementary Materials

The following supporting information can be downloaded at: https://www.mdpi.com/article/10.3390/ijms27020976/s1.

## Abbreviations

ACIN1	Apoptotic Chromatin Condensation Inducer 1
ANOVA	Analysis of Variance
ANXA1	Annexin A1
ANXA6	Annexin A6
APO-BrdU TUNEL Assay	Apoptosis detection by DNA fragmentation
ATP	Adenosine Triphosphate
ApoBDs	Apoptotic Bodies
BCA	Bicinchoninic Acid
BrdUTP	5-Bromo-2′-deoxyuridine 5′-Triphosphate
CASP-1	Caspase-1
DMSO	Dimethyl Sulfoxide
EM	Electron Microscopy
EVs	Extracellular Vesicles
FBS	Fetal Bovine Serum
GSDMD	Gasdermin D
IL-18	Interleukin-18
IL-1β	Interleukin-1 beta
lEVs	Large Extracellular Vesicles
LPS	Lipopolysaccharide
MCC950	Specific NLRP3 Inhibitor
MISEV	Minimal Information for Studies of Extracellular Vesicles
MS	Mass Spectrometry
NIG	Nigericin
NLRP3	NOD-, LRR- and pyrin domain-containing protein 3
NTA	Nanoparticle Tracking Analysis
PANoptosis	Pyroptosis, Apoptosis, and Necroptosis
PBS	Phosphate-Buffered Saline
PI	Propidium Iodide
PFA	Paraformaldehyde
PPI	Protein–Protein Interaction
Q-VD-OPh (QVD)	Pan-Caspase Inhibitor
RCD	Regulated Cell Death
RLU	Relative Light Units
RPMI-1640	Roswell Park Memorial Institute Medium
SPV	Sulfo-Phospho-Vanillin (Lipid Assay)
sEVs	Small Extracellular Vesicles
STS	Staurosporine
TdT	Terminal Deoxynucleotidyl Transferase
∆ψm	Mitochondrial Membrane Potential
