# Supplementary material for: Characterization of Large Extracellular Vesicles Released by Apoptotic and Pyroptotic Cells
Source: Int J Mol Sci. 2026 Jan 19;27(2):976. doi: 10.3390/ijms27020976 (PMC12841904; doi:10.3390/ijms27020976)
Supplement: Supplementary file 1 [file ijms-27-00976-s001.zip › ijms-3904476-supplementary.pdf]

Supplementary Material

Characterization of large extracellular vesicles released by apoptotic and pyroptotic cells

Delaram Khamari [1,2], Nora Fekete [1], Ririka Tamura [1], Agnes Kittel [3], Raeeka Khamari [4], Bence Nagy[1,2], Alicia Galinsoga[1,2], Zsuzsanna Darula[5,6], Eva Hunyadi-Gulyas[6], Maximilien Bencze[*7§], Edit I Buzas [*1,2,3§]

[1]Institute of Genetics, Cell- and Immunobiology, Semmelweis University, Budapest, Hungary
[2]HUN-REN-SU Translational Extracellular Vesicle Research Group, Budapest, Hungary
3HUN-REN, Institute of Experimental Medicine, Budapest, Hungary
[4] Lille University Hospital (CHU), Henri Warembourg Faculty of Medicine, Lille, France
[5] Single Cell Omics Advanced Core Facility, Hungarian Centre of Excellence for Molecular Medicine, Szeged, Hungary
[6] Proteomics Research Group, Core Facility, HUN-REN Biological Research Centre, Szeged, Hungary
[7] U955-IMRB, Inserm, UPEC, ENVA, EFS, Team Relaix, Biology of the Neuromuscular System, Créteil 94000, France

Supplementary Table S1

Reagent	Company	Catalogue #	Notes / Application
**Cell culture reagents**			
**RPMI-1640**	Sigma-Ald, St. Louis, MO, USA	R6504	Cell culture medium
**FBS**	Sigma-Ald, St. Louis, MO, USA	F9665	Supplement for medium
**Penicillin–Streptomycin**	Gibco, Thermo Fischer, Waltham, Massachusetts, US	15140-122	Antibiotic
**Sodium Pyruvate**	Sigma-Ald, St. Louis, MO, USA	S8636	Supplement
**HEPES**	Gibco, Thermo Fischer, Waltham, Massachusetts, US	15630-080	Buffer
**DMSO**	Sigma-Ald, St. Louis, MO, USA	D2650	Solvent
**Apoptosis / Pyroptosis /**			
**Staurosporine**	Sigma-Ald, St. Louis, MO, USA	S4400	Apoptosis inducer
**Q-VD-OPh**	Sigma-Ald, St. Louis, MO, USA	551476	Pan-caspase inhibitor
**Lipopolysaccharide (LPS)**	Sigma-Ald, St. Louis, MO, USA	L3129	Inflammasome activation
**Nigericin**	InvivoGen	tlrl-nig	NLRP3 activation
**NLRP3 inhibitor**	Sigma-Ald, St. Louis, MO, USA	538120	Inflammasome inhibition
**Assay kits**			
**CellTiter-Glo®**	Promega, Budapest, Hungary	G7571	Cell viability
**APO-BrdU™ TUNEL Assay Kit, with Alexa Fluor™ 488 Anti-BrdU**	Invitrogen, Carlsbad, CA, USA),	A23210	DNA fragmentation
**CellEvent™ Caspase-3/7 Green Flow Cytometry Kit**	Invitrogen, Carlsbad, CA, USA),	C10427	Caspase-3/7 activity
**Caspase-1 (Active) Staining Kit, Green**	Abcam	ab219935	Caspase-1 activity
**Cell Meter™ Live Cell Caspase-1 Binding Assay, Green**	AAT Bioquest	20108	Caspase-1 activity
**Human IL-1β ELISA Kit**	Sigma-Ald,St. Louis, MO, USA	RAB0273-1KT	Cytokine measurement
**Antibodies**			
**Annexin A6 antibody**	Santa Cruz Biotechnology	F2521	Detection of Annexin A6
**Propidium iodide**	Sigma	P4864	Apoptosis/necrosis staining
**Annexin V**	Santa Cruz Biotechnology	F2521	Apoptosis/necrosis staining
**CD9 monoclonal antibody**	Sony	162120	EV/exosome marker
**CD63 monoclonal antibody**	Sony	195923	
**CD81 monoclonal antibody**	Sony	158543	EV/exosome marker

Supplementary Figure S1 : Comparative analysis of protein and lipid content of EVs

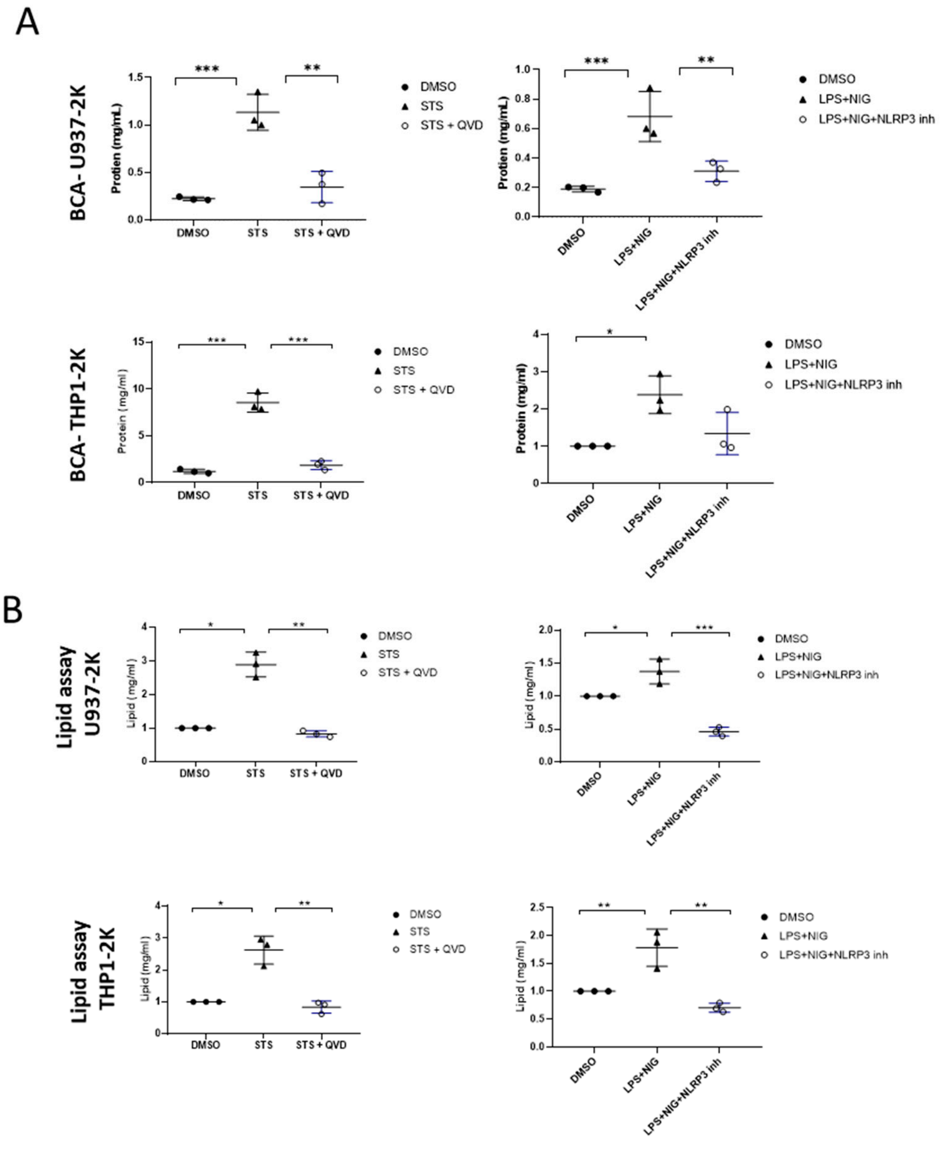

(A) Bicinchoninic acid (BCA) protein assay of 2K and 12.5K pellet under control, LPS+NIG, and STS conditions.

(B) Lipid quantification of 2K and 12.5K pellet using a lipid assay. Data are presented as mean ± SEM from independent experiments (n = 3). **p< 0.05,* ***p< 0.01,* ****p< 0.001* (one-way ANOVA).

Supplementary figure S2 : Gating strategy for nanoparticle detection using CytoFLEX S flow cytometer

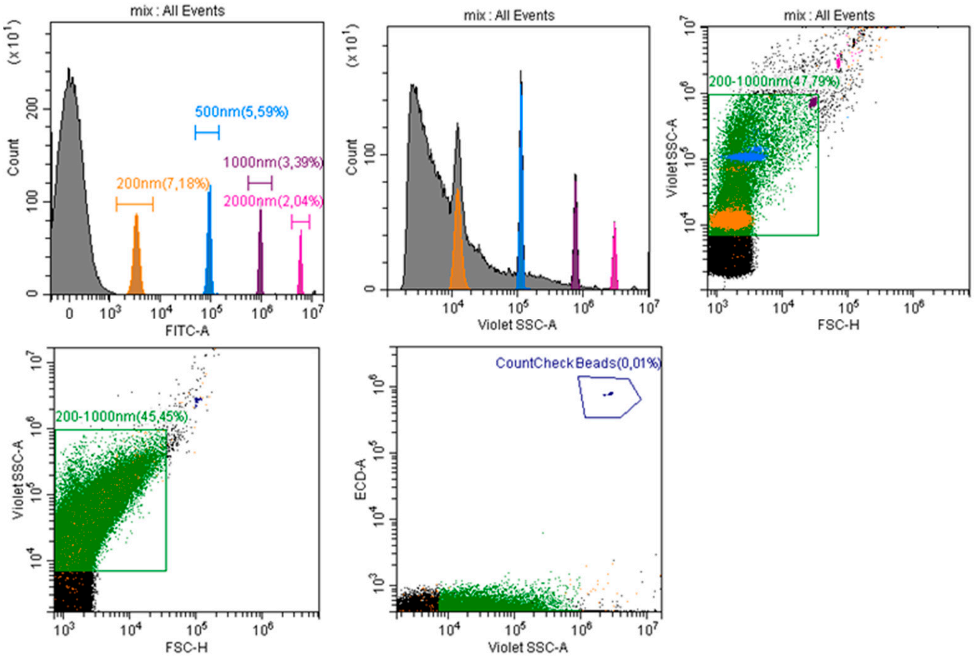

Representative flow cytometry plots showing the discrimination of fluorescent polystyrene bead populations of defined sizes (200 nm, 500 nm, 1000 nm, 2000 nm) and CountCheck beads. (Top left) FITC-A histogram used to identify the 200 nm, 500 nm, 1000 nm, and 2000 nm beads based on fluorescence intensity. (Top middle) Violet SSC-A histogram showing clear resolution of bead populations by side scatter. (Top right) FSC-H versus Violet SSC-A dot plot with gates used to define nanoparticles in the 200–1000 nm range. (Bottom left) FSC-H versus Violet SSC-A confirming the nanoparticle gate. (Bottom right) Violet SSC-A versus ECD-A dot plot showing the CountCheck beads used for standardization and event quantification. The percentage of each gated population is indicated on the plots.

Supplementary Figure S3. Proteomic analysis of protein expression changes in response to pyroptosis and apoptosis induction

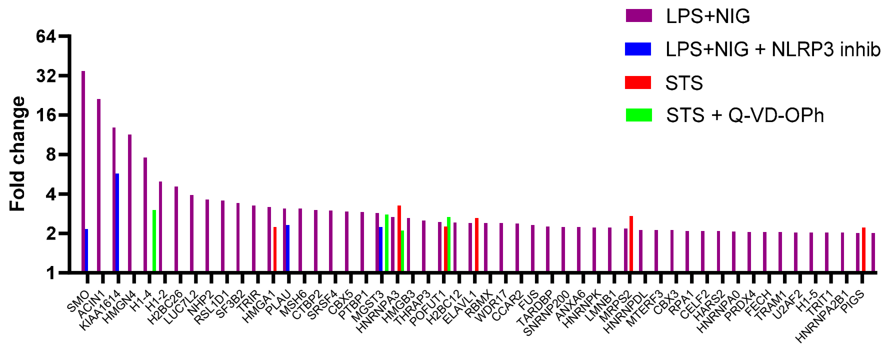

Mass spectrometry (MS)-based quantification of proteins showing differential regulation following stimulation with LPS + nigericin (LPS+NIG, purple), LPS+NIG in the presence of an NLRP3 inhibitor (blue), staurosporine (STS, red), or STS with pan-caspase inhibitor Q-VD-OPh (green). Bars represent fold change relative to the untreated control. Data are presented as fold change (y-axis) for selected proteins (x-axis).

Supplementary Table S2: Relative abundance of proteins (referred to the control) in the 2K and 12.5K pellets upon distinct cell death stimuli and available related published data

2K pellet of THP1 cells

Protein in 2K pellet	Description	Ratio: (LPS+NIG) / (CTRL)	Abundance Ratio: (LPS+NIG+NLRP3 inh) / (CTRL)	Abundance Ratio: (STS) / (CTRL)	Ratio: (STS_QVD) / (CTRL)
TBXAS1	Thromboxane synthase; prostanoid biosynthesis				2.261
LGALS8	Galectin-8; lectin sensing damaged vesicles				2.695
YWHAG	14-3-3 protein θ; signaling adapter		0.489		
CSTB	Cystatin B; cysteine protease inhibitor	0.482	0.429		
FABP5	Fatty acid-binding protein	0.491			
WDR1	Actin cytoskeleton remodeling factor	2.401			
SH3BGRL	SH3 domain-binding protein	0.474	0.481		
MT1E	Metallothionein	0.447	0.437		
ALDOA	Aldolase A; glycolysis enzyme	0.473	0.485		
LDHA	Lactate dehydrogenase A	0.465	0.496		
ARHGDIB	Rho GDP dissociation inhibitor β	0.472			
PEA15	Death effector domain protein	0.491			
NRBP1	Kinase regulator				2.614
MIF	Macrophage migration inhibitory factor	0.445	0.486		
TBCA	Tubulin-folding cofactor A	0.38	0.415		
LNPPS	Long non-coding RNAs				
POFUT1	Protein O-fucosyltransferase 1	2.434		2.258	2.669
HNRNPA0	heterogeneous ribonucleoprotein (hnRNP) A0	2.071			
ELAVL1	ELAV-like protein 1 or HuR (human antigen R)	2.404		2.632	
CTSS	Cathepsin S Lysosomal protease for antigen processing				0.499
POTEE	POTE Ankyrin Domain Family Member E		2.063		
MEFV	Pyrin is a cytosolic protein	0.413		0.437	
PTBP1	polypyrimidine tract-binding protein 1	2.9			
HNRNPA2B1	RNA-binding proteins	2.018			
H1-2	Histone variants	4.949			
HMGB3	High-mobility group box 3	2.618			
HNRNPK	Heterogeneous nuclear ribonucleoprotein K	2.219			
ADAM10	metallopeptidase domain 10	2.003			
FUS	FUS RNA Binding Protein	2.317			
HMGA1	High Mobility Group AT-Hook 1	3.19		2.233	
ANXA6	Annexin A6	2.229			
RBMX	RNA binding motif protein X-linked	2.403			
SMO	Smoothened, Frizzled Class Receptor)	34.61	2.151		
PLAU	Plasminogen Activator, Urokinase	3.105	2.316		
HDAC7	histone deacetylase 7		2.03		
CYCS	cytochrome c	0.494		0.384	

Protein in 12.5K pellet	Description	Abundance Ratio: (LPS+NIG) / (CTRL)	Abundance Ratio: (LPS+NIG+ NLRP3 inh) /(CTRL)	Abundance Ratio: (STS) / (CTRL)	Abundance Ratio: (STS_QVD) /(CTRL)
ELAVL1	RNA-binding protein (mRNA stability regulator)			2.969	
LGALS8	Galectin (lectin)	2.856			
ANXA1	Annexin (membrane repair / anti-inflammatory roles)	3.114			
LGALS3	Galectin (lectin)	5.195			
ANXA5	Annexin (phospholipid binding)				2.059
CHMP4B	ESCRT-III component				2.004
VASH2	Angiogenesis regulator (tubulin carboxypeptidase)	0.265			
SOD1	Superoxide dismutase (oxidative stress)	0.34			
CHMP5	ESCRT component / endosomal sorting	0.095			
RAB5A	Early endosome GTPase (vesicle trafficking)	0.082			
RPL35A	Ribosomal protein	0.163			
SLC8A1	Na+/Ca2+ exchanger	0.263			
DDAH2	Nitric oxide regulation (ADMA metabolism)	0.26			
GOLGA3	Golgi matrix protein	0.333			
CXCR4	Chemokine receptor (cell migration)	0.174			
MBP	Myelin basic protein	0.178			
PEA15	Death-effector domain protein (apoptosis signaling)	0.303			
HNRNPC	Heterogeneous nuclear ribonucleoprotein	0.249			
FLG	Filaggrin (epidermal protein)	0.293			
GNG10	G protein gamma subunit	0.305			
PLEKHO2	Pleckstrin homology domain protein	0.053			
EZR	Ezrin (membrane–cytoskeleton linker)	0.373			
HMGA1	Chromatin architectural factor				
PIP	Plasma protease inhibitor	0.153		0.32	
AZGP1	Lipid-binding protein (adipokine)	0.278		0.215	
KRT6A	Keratin	0.321		0.183	0.327
KRT14	Keratin	0.362		0.258	0.355

Proteins enriched in the 2K and 12.5K pellets were identified and quantified under various conditions: LPS + nigericin (LPS+NIG), LPS+NIG with NLRP3 inhibition, staurosporine (STS), and staurosporine with Q-VD-OPh (STS_QVD). Abundance ratios relative to untreated control (CTRL) are shown for each condition. Published associations of each protein with apoptosis or pyroptosis are indicated, with corresponding reference numbers listed.

Supplementary Figure S4.

Induction of apoptosis and pyroptosis of HeLa cells

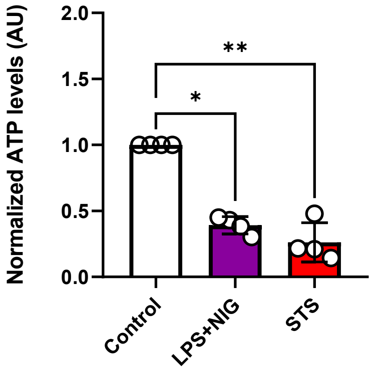

Cell viability of HeLa cells measured by CellTiter-Glo assay. Cells (untreated controls, or treated with either LPS+NIG or STS) were analyzed. Data are represented as mean ± SEM (n = 4). *$p< 0.05$, **$p< 0.01$ (one-way ANOVA, uncorrected Dunn's test).

Supplementary Figure S5.

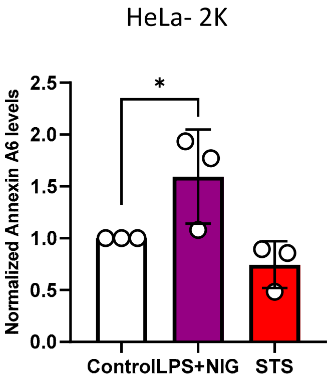

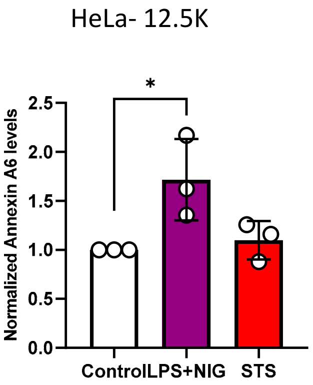

Annexin A6 expression of lEVs released from HeLa cells undergoing apoptosis and pyroptosis determined by flow cytometry. Data are represented as mean ± SEM (n = 3). *p< 0.05 (one way ANOVA)

Supplementary Figure S6.

Annexin V/PI positivity of monocyte cell lines exposed to pyroptotic and apoptotic EVs

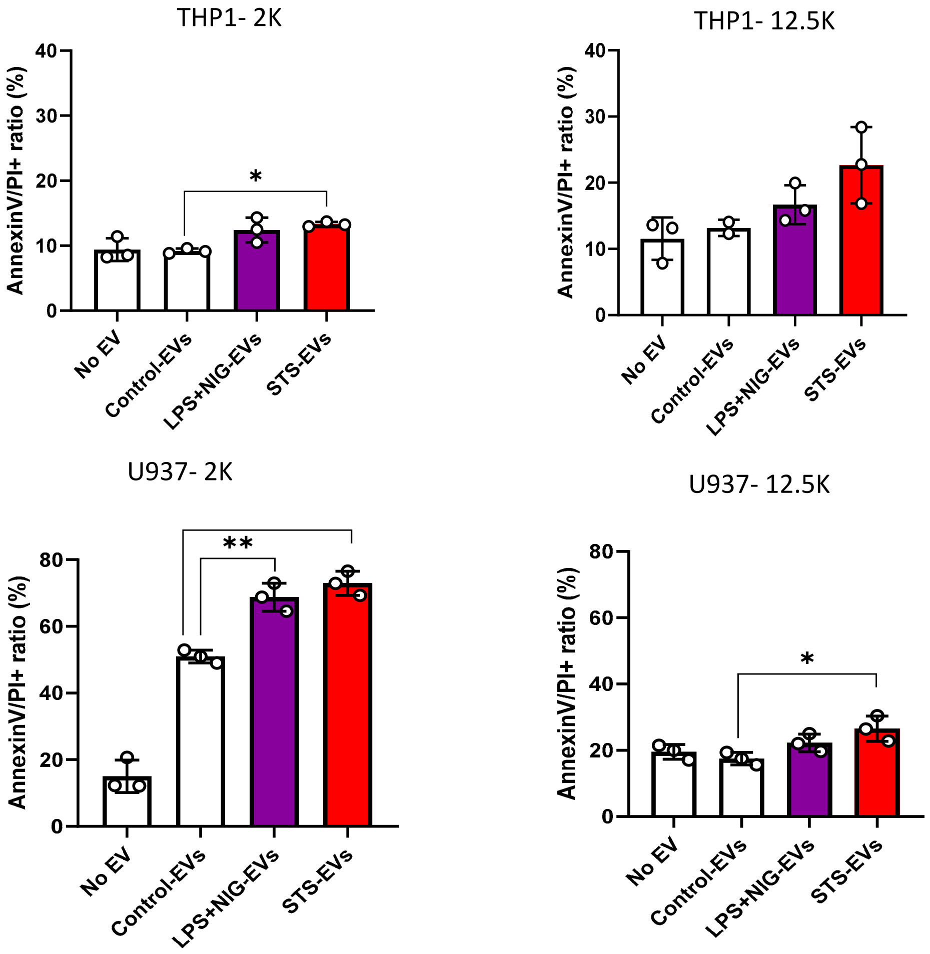

Annexin V/PI positivity of THP1 and U937 cells treated with apoptotic and pyroptotic EVs. THP1 and U937 were cultured in 24 well plates (150,000 cells/well) in the presence or absence of 10^4 EVs/cell for 24 hours in 10% EV-depleted FBS. Data are represented as mean ± SEM (n = 3). *$p< 0.05$, **$p< 0.01$ (one-way ANOVA).

Supplementary Figure S7.

IL-1β levels in the conditioned media of monocytic cell lines upon exposure to pyroptotic or apoptotic EVs

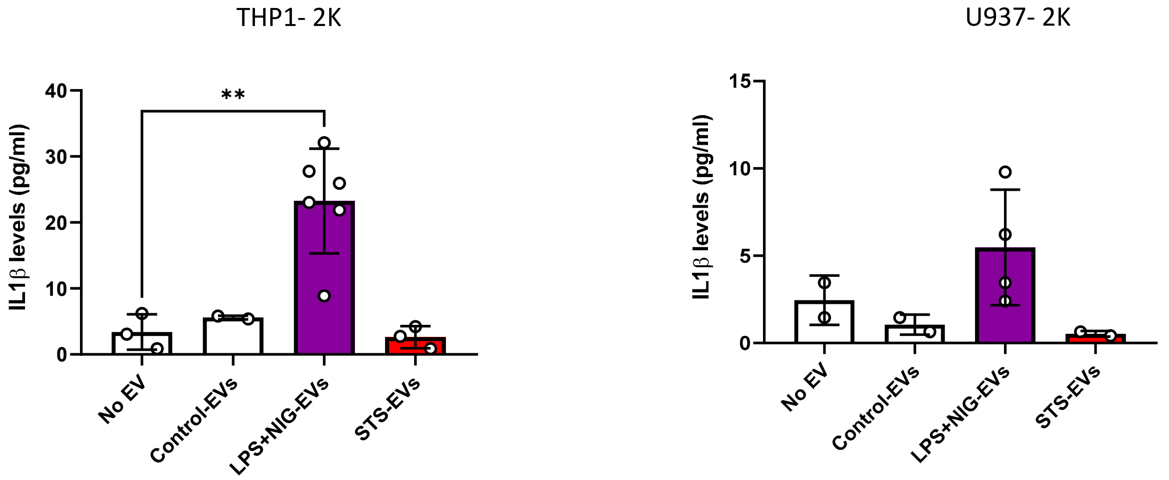

THP1 and U937 were cultured in 24 well plates (150,000 cells/well) in the presence or absence of 10^4 EVs/cell for 24 hours in 10% EV-depleted FBS. The conditioned medium was collected after 24 hours, and the IL-1β concentration was determined using an ELISA kit (n = 3). ***p< 0.01* (one-way ANOVA).

Supplementary Figure S8.

Expression of tetraspanins on the surface of the EV releasing cells

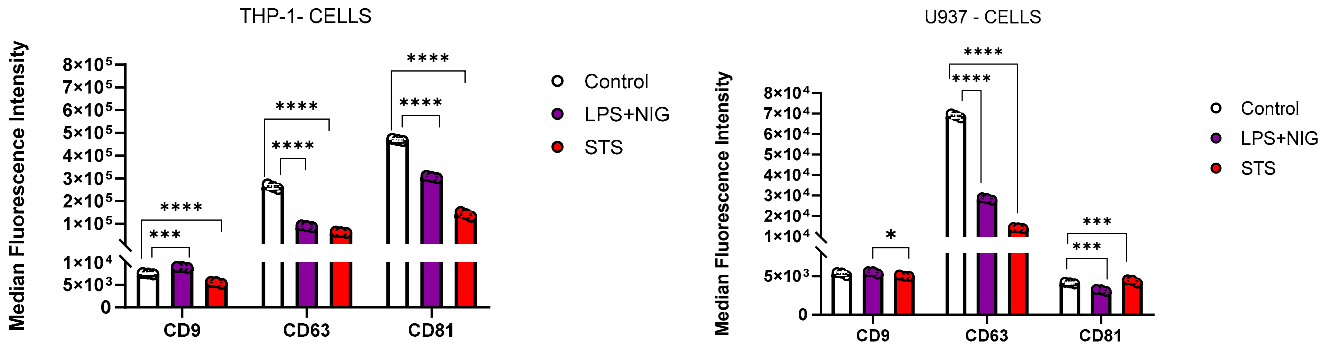

Cell surface expression of the canonical EV surface tetraspanins was also determined in the case of the EV releasing cells showing different expression patterns as compared to the released vesicles (n=3). **p< 0.05*, ****p< 0.001*, *****p< 0.0001* (one-way ANOVA).
